# Multi-Omics Sequencing Provides Insights Into Age-Dependent Susceptibility of Grass Carp (*Ctenopharyngodon idellus*) to Reovirus

**DOI:** 10.3389/fimmu.2021.694965

**Published:** 2021-06-17

**Authors:** Libo He, Denghui Zhu, Xinyu Liang, Yongming Li, Lanjie Liao, Cheng Yang, Rong Huang, Zuoyan Zhu, Yaping Wang

**Affiliations:** ^1^ State Key Laboratory of Freshwater Ecology and Biotechnology, Institute of Hydrobiology, Chinese Academy of Sciences, Wuhan, China; ^2^ College of Advanced Agricultural Sciences, University of Chinese Academy of Sciences, Beijing, China; ^3^ Innovative Academy of Seed Design, Chinese Academy of Sciences, Beijing, China

**Keywords:** grass carp, age-dependent viral susceptibility, grass carp reovirus, immune response, biosynthesis, metabolism

## Abstract

Grass carp (*Ctenopharyngodon idellus*) is an important aquaculture species in China that is affected by serious diseases, especially hemorrhagic disease caused by grass carp reovirus (GCRV). Grass carp have previously shown age-dependent susceptibility to GCRV, however, the mechanism by which this occurs remains poorly understood. Therefore, we performed transcriptome and metabolome sequencing on five-month-old (FMO) and three-year-old (TYO) grass carp to identify the potential mechanism. Viral challenge experiments showed that FMO fish were susceptible, whereas TYO fish were resistant to GCRV. RNA-seq showed that the genes involved in immune response, antigen presentation, and phagocytosis were significantly upregulated in TYO fish before the GCRV infection and at the early stage of infection. Metabolome sequencing showed that most metabolites were upregulated in TYO fish and downregulated in FMO fish after virus infection. Intragroup analysis showed that arachidonic acid metabolism was the most significantly upregulated pathway in TYO fish, whereas choline metabolism in cancer and glycerophospholispid metabolism were significantly downregulated in FMO fish after virus infection. Intergroup comparison revealed that metabolites from carbohydrate, amino acid, glycerophospholipid, and nucleotide metabolism were upregulated in TYO fish when compared with FMO fish. Moreover, the significantly differentially expressed metabolites showed antiviral effects both *in vivo* and *in vitro*. Based on these results, we concluded that the immune system and host biosynthesis and metabolism, can explain the age-dependent viral susceptibility in grass carp.

## Introduction

Grass carp (*Ctenopharyngodon idellus*) is an important aquaculture species in China, accounting for more than 18% of the total freshwater aquaculture production in the country. Production of grass carp reached 5.53 million tons in 2019, making it the most highly consumed freshwater fish worldwide (1). However, grass carp is susceptible to many pathogens, especially the grass carp reovirus (GCRV), which causes grass carp hemorrhagic disease, a significant threat to the aquaculture of the species (2, 3). Consequently, GCRV has received much attention from fish breeding scientists and fish immunologists who aim to achieve disease-resistant breeding and uncover the mechanism underlying GCRV infection (4–6). Grass carp have shown age-dependent susceptibility to GCRV, with those less than 1 year old being susceptible to GCRV, while those over 3 years of age being resistant to the virus (7). However, the mechanism by which this occurs remains poorly understood.

In mammals, several studies on age-dependent susceptibility to viral infections have been done. Pott et al. (8) showed that neonatal mice were susceptible to rotavirus (8), whereas no clinical signs of infection were observed in adult individuals. This suggests that age-dependent TLR3 expression in the intestinal epithelium contributes to rotavirus susceptibility. In humans, infants and young children are particularly susceptible to viral encephalitis, whereas adults are not. A mouse model-based study revealed that age-dependent susceptibility to reovirus encephalitis was determined by the maturation of type-I interferon response (9). In fish, age is also an important factor that influences susceptibility to viruses. For example, rainbow trout fry showed a decrease in susceptibility to infectious pancreatic necrosis virus (IPNV) with increasing age and ceased to be susceptible at 20 weeks of age (10). Spring viremia of carp virus (SVCV) challenge experiments in multiple North American fish species suggested that host age is a key factor in determining disease outcomes (11). Age-dependent susceptibility to nervous necrosis virus (NNV) was also demonstrated in barramundi (*Lates calcarifer*) whereby infection at 3 and 4 weeks of age caused nervous necrosis disease, while fish at 5, 7, and 9 weeks of age developed subclinical infection (12). However, the mechanism underlying age-dependent susceptibility to viral infections in fish is largely unknown.

For the fish that showed age-dependent susceptibility to virus infection, the younger fish and older fish were considered as sensitive and resistant groups, respectively. Therefore, comparing two groups by specific methods may reveal the key genes or pathways involved in this phenomenon and provide important information for disease-resistant breeding or disease control and prevention. Moreover, the results could also provide useful information on age-dependent viral diseases in humans. In this study, we used five-month-old (FMO) and three-year-old (TYO) grass carp as the sensitive and resistant groups for further study. The age-dependent susceptibility to GCRV in grass carp was confirmed by an artificial viral challenge experiment. Additionally, histopathology, transcriptomics, and metabolomics were employed to uncover the mechanisms underlying this phenomenon. Due to the details of molecular events following GCRV infection have been reported previously (13); therefore, the main aim of the current study was not to identify the genes or pathways associated with GCRV infection again, but to reveal the potential mechanism of age-dependent viral susceptibility in grass carp by comparing susceptible FMO fish with resistant TYO fish. The results will improve our understanding of age-dependent susceptibility to virus infection in grass carp and will benefit disease-resistant breeding programs or GCRV control and prevention.

## Materials and Methods

### Experimental Animals, Virus Exposure, and Sample Collection

**Figure 1:**
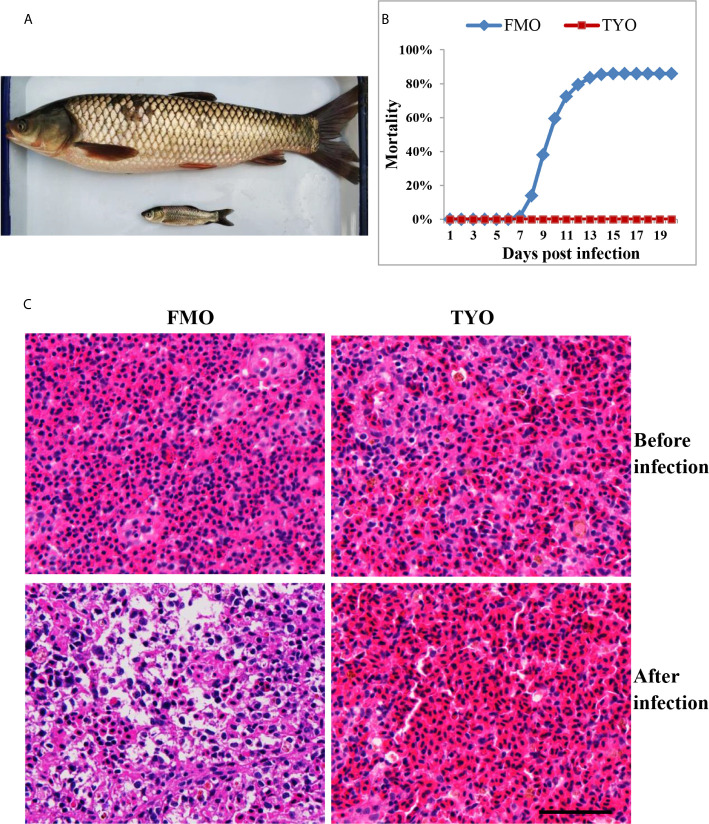
The age-dependent susceptibility to GCRV in grass carp. **(A)** The representative picture of FMO grass carp and TYO grass carp. **(B)** The cumulative mortality of two groups of grass carp after GCRV infection. The total mortality of FMO fish group was reached to 86% while no dead fish was observed in the TYO fish group. **(C)** Histological section observation of two fish groups before and after GCRV infection. The spleen samples from two groups that that collected before and after (7 days post infection) GCRV infection were subjected histological section preparation and observed under microscope. Scale bars = 100 um.

Approximately 300 FMO and 300 TYO grass carp were used in the study. FMO fish had an average weight and length of 8 g and 12 cm, respectively, while the average weight and length of TYO fish were 2–3 kg and 50 cm, respectively (**Figure 1A**). All fish were obtained from the Guan Qiao Experimental Station, Institute of Hydrobiology, Chinese Academy of Sciences (CAS), and acclimated in aerated fresh water at 26–28°C for one week before processing. Fish were fed commercial feed twice daily, and water was exchanged daily. If no abnormal symptoms were observed, the fish were selected for further study.

After no abnormal symptoms were observed in the two groups, a viral challenge experiment was performed. Fish were infected with GCRV (GCRV subtype II, 2.97 × 10^3^ RNA copies/µL) at a dose of 20 µL per gram of body weight by intraperitoneal injection. At the time before injection (0 days) and 1-5 days after injection, 15 fish from each group were anesthetized and euthanized with MS-222 (100 mg/L), and the spleens were removed for analysis. The collected samples were used for RT-qPCR analysis, histological section preparation, and transcriptome and metabolome sequencing. The remaining fish were carefully monitored, and the number of daily deaths was recorded. The experiment was concluded when no mortality was recorded for seven consecutive days, and the overall mortality rate could be calculated.

### Histopathology

Histological sections were prepared as described previously (14). Briefly, spleen samples from three individuals of each group that were collected at 0, 1, 3, 5, and 7 days post-infection (dpi) were fixed in 4% paraformaldehyde overnight at 4°C. Following dehydration, the samples were embedded in HistoResin (Leica). Serial sections of 4 μm thickness were cut using a microtome (Leica), dried on slides at 42°C overnight, stained with hematoxylin and eosin (H&E), mounted in Permount (Fisher), and imaged with phase contrast with a 100× oil immersion objective lens.

### Transcriptome Sequencing

RNA was isolated from the spleens using TRIzol reagent (Invitrogen, USA) according to the manufacturer’s protocol. Sequencing libraries were generated using the NEBNext Ultra RNA Library Prep Kit for Illumina (New England Biolabs, USA) following the manufacturer’s protocol. Libraries were sequenced on an Illumina Novaseq platform, and 150 bp pair-end reads were generated. The output raw data reads were processed as described previously to obtain clean data (13). The clean reads were mapped to the reference genome of grass carp using Hisat2 software (15), and gene expression levels were calculated by FPKM (expected number of fragments per kilobase of transcript sequence per million base pairs sequenced) methods (16).

Differential expression analysis of the two groups/conditions was performed using the DESeq package (17). The resulting P-values were adjusted using the Benjamini and Hochberg approach to control the false discovery rate. Genes with an adjusted P-value < 0.05 (q value < 0.05) in DESeq analysis were assigned as differentially expressed genes (DEGs). All the DEGs identified in this study were used as references for the Gene Ontology (GO) and Kyoto Encyclopedia of Genes and Genomes (KEGG) enrichment analysis using the GOseq R package and KOBAS software (18, 19).

### Examining the Expression Patterns of Immune Related Genes by RT-qPCR

Eight genes involved in the immune response were selected for RT-qPCR analysis to confirm the reliability of the RNA-seq data. Spleen samples from the two groups before and after GCRV infection were obtained, and RNA samples were prepared. First-strand cDNA was obtained using a random hexamer primer and ReverTra Ace kit (Toyobo, Japan). RT-qPCR was performed using a fluorescence quantitative PCR instrument (Bio-Rad, USA). Each RT-qPCR mixture contained 0.8 μL forward and reverse primers (for each primer), 1 μL template, 10 μL 2× SYBRgreen master mix (TOYOBO, Japan), and 7.4 μL ddH2O. Three replicates were included for each sample, and the β-actin gene was used as an internal control for normalization of gene expression. The relative expression levels of genes in the TYO group were calculated as the ratio of gene expression levels relative to those in the FMO group at the corresponding time point. The primers are listed in **Table S1**. The RT-qPCR program was as follows: 95°C for 10 s, 40 cycles of 95°C for 15 s, 55°C for 15 s, and 72°C for 30 s, followed by melt curve construction. Relative expression levels were calculated using the 2^-△△Ct^ method (20). Data represent the mean ± standard deviation of three replicates.

### Extraction of Metabolites

The spleen samples collected were frozen immediately in liquid nitrogen and then preserved at − 80°C. For metabolite extraction, the samples were thawed slowly on ice. Samples (50 mg) were homogenized with 1000 μL of ice-cold methanol/water (70%, v/v) using an Ultra-Turrax homogenizer. Cold steel balls were added to the mixture and homogenized at 30 Hz for 3 min. The mixtures were stirred for 1 min, centrifuged at 12,000 rpm at 4°C for 10 min, and the collected supernatant was used for further analysis.

### UPLC -MS/MS Analysis

Metabolites were determined *via* ultra-performance liquid chromatography-tandem mass spectrometry (UPLC-MS/MS), as described previously (21, 22). Briefly, the UPLC system (Shim-pack UPLC SHIMADZU CBM30A) combined with MS/MS (QTRAP 6500+) was set at 30,000 resolution to obtain UPLC-MS/MS statistics. Sample analysis was performed in positive ion modes, a spray voltage of 5.5 kV, negative ion modes, spray voltage of -4.5 kV, and capillary temperature of 500°C. The mass scanning scope was set from 50 to 1,500 m/z. The nitrogen sheath and nitrogen auxiliary gas were set at 30 L/min and 10 L/min, respectively. Solvent A was 0.04% acetic acid (Fisher Scientific)/water (Millipore) (v/v), and solvent B was 0.04% acetic acid/acetonitrile (Fisher Scientific) (v/v). The gradient flow rate was 0.4 mL/min and the column temperature was 40°C, and the process was as follows: 5% B at 0 min, 95% B at 11.0 min, 95% B at 12.0 min, 5% B at 12.1 min, and 5% B at 14 min. The QC samples were injected four times at the start to ensure system consistency. A Waters ACQUITY UPLC HSS T3 C18 column (100 × 2.1 mm, 1.8 μm) was used for all analyses.

### Metabolite Identification and Data Processing

Metabolite identification was based on the primary and secondary spectral data annotated against the self-compiled database MWDB (WuhanMetware Biotechnology Co., Ltd.) and publicly available metabolite databases, including MassBank (http://www.massbank.jp/), KNApSAcK (http://kanaya.naist.jp/KNApSAcK/), HMDB (http://www.hmdb.ca/), MoToDB (http://www.ab.wur.nl/moto/), and METLIN (http://metlin.scripps.edu/index.php). Metabolite quantification was performed using the multiple reaction monitoring (MRM) mode (23). Orthogonal partial least squares discrimination analysis (OPLS-DA) was used to study the identified metabolites. Those with significant differences in content were set with thresholds of variable importance in projection (VIP) ≥ 1 and | Log2fold change | ≥ 1.

### CCK-8 Assay

A CCK-8 detection kit (Beyotime, Shanghai, China) was used to investigate the effects of the metabolites on cell viability according to the manufacturer’s instructions. Briefly, approximately 5 × 10^3^ *Ctenopharyngodon idellus* kidney (CIK) cells were seeded in 96 well plates and cultured in M199 medium supplemented with 10% fetal bovine serum (FBS) at 28°C for 24 h. Cells were treated with metabolites at different concentrations for 24 h. Then, 10 µL of CCK-8 solution was added to each well and incubated at 28°C for 4 h, and the absorbance at 450 nm was measured using a microplate reader (BIO-RAD, Hercules, CA, USA). The untreated cells were considered as the positive control, while the wells containing no cells but only culture medium were used as blank controls. The data are represented as the mean ± standard deviation (SD) values of three replicates.

### Investigating the Anti-Viral Effects of Significantly Dems *In Vitro* and *In Vivo*

CIK cells were seeded in 6-well plates and grown until they formed a monolayer with 90% confluency. Before GCRV infection, the medium was replaced with a metabolite-supplemented medium at different concentrations and incubated for 4 h. Cells were then infected with GCRV at a multiplicity of infection (MOI) of 0.1 and harvested at 24 h post infection. The copy numbers of non-structural protein NS80 and structural protein VP7 were determined by RT-qPCR as described above. Additionally, plaque assays were performed to investigate the antiviral effects of the metabolites. Briefly, the infected cells in 12-well plates were overlaid with a medium containing 0.7% melted soft agar. After 24–48 h post-infection, the plaques formed and the medium was removed. The cells were then fixed with 20% formaldehyde and stained with 1% crystal violet. Three biological duplications were performed for the plaque assays and therefore the statistic data of the plaques in different groups were calculated and compared.

Approximately 400 FMO grass carp were randomly divided into four groups, one hundred each. The fish were then intraperitoneally injected with different metabolites (arachidonic acid: 100 μM; L-tryptophan: 5 mM; adenosine: 500 μM) at a volume of 200 μL or the same volume of PBS (control group), once daily for 3 days. After that, a viral challenge experiment was carried out for these fish by intraperitoneal injection as described above. The experiment was concluded when no mortality was recorded for seven consecutive days, and the total mortality in each group was calculated.

### Statistical Analysis

The statistical significance between different groups was determined by one-way analysis of variance (ANOVA) and Fisher’s least significant difference (LSD) posttest. Differences were considered significant at P < 0.05. P < 0.05 was denoted by *.

## Results

### Age-Dependent Susceptibility to GCRV in Grass Carp

Representative images of FMO and TYO grass carp are shown in **Figure 1A**. A viral challenge was performed for FMO and TYO grass carp. **Figure 1B** shows that a mortality rate of 86% in the FMO fish group was reached at 15 days after infection with GCRV, with the first death recorded 8 days post-infection (dpi). In contrast, no dead fish were observed in the TYO fish group. Histological sections from both groups showed no visible difference between spleen samples before GCRV infection; cells in both groups had an orderly arrangement, and the nuclei were intact (**Figure 1C**). However, the post-infection spleen samples from FMO fish showed severe necrotic lesions, vacuolization, and hypertrophied nuclei with karyorrhexis, while no obvious change was observed in the spleen samples from TYO fish. Therefore, these results further confirm age-dependent susceptibility to GCRV in grass carp.

### Transcriptome Analysis of Grass Carp With Different Ages Before and After Viral Challenge

To further elucidate the mechanism of age-dependent susceptibility to GCRV in grass carp, we performed RNA-seq analysis on samples collected from the two age groups before (0 d) and after (1, 3, and 5 d) infection. The samples in the FMO group were named S1-0, S1-1, S1-3, and S1-5, while samples in the TYO group were named as S3-0, S3-1, S3-3, and S3-5. Three duplicates of each sample were processed, yielding a total of 24 libraries, which were sequenced on an Illumina Novaseq platform to generate 150 bp pair-end reads. In total, each library yielded clean bases ≥ 6 GB, Q20 ≥ 95%, Q30 ≥ 87%, and uniquely mapped percentage ≥ 85% (**Table S2**), confirming the high quality of the sequence data and its suitability for further analysis. The sequence data from this study were deposited in the Sequence Read Archive (SRA) at the National Center for Biotechnology Information (NCBI) (accession number: PRJNA600033). These data were subjected to a series of intergroup comparisons to identify the DEGs. Briefly, data from the TYO fish group (S3-0, S3-1, S3-3, and S3-5) were compared with data from the FMO fish group (S1-0, S1-1, S1-3, and S1-5) at the same time points. In detail, 300, 898, 393, and 428 DEGs were upregulated, whereas 569, 1040, 555, and 724 DEGs were downregulated at 0, 1, 3, and 5 dpi, respectively (**Table S3**). Detailed information on these DEGs is presented in **Table S4**.

### Enrichment Analysis of the DEGs

GO and KEGG enrichment analyses were used to identify the specific roles of the DEGs. To discriminate the virus infection process in fish between the different groups, the upregulated and downregulated DEGs from each time point were separately subjected to enrichment analysis. As shown in **Table 1**, before GCRV infection (0 d), GO enrichment analysis showed that the upregulated DEGs were significantly enriched with terms involved in proteasome and metal ion homeostasis, while the downregulated DEGs were mainly enriched with terms related to enzyme activity. At 1 and 3 dpi, GO terms involved in immune response, antigen presentation, and chemokine and cytokine activity were significantly enriched for upregulated DEGs, whereas terms responsible for enzyme activity and the oxidation-reduction process were enriched for downregulated DEGs. Finally, binding-related GO terms were enriched for the upregulated DEGs from 5 dpi, whereas chemokine and cytokine activity-related terms were enriched for the downregulated DEGs. The results support that the immune response of TYO fish started immediately, while in FMO fish it was activated slowly. The top five enriched GO terms (up and downregulated) at each time point are listed in **Table 1** and full details of the GO terms are given in **Table S5**.

**Table 1:** The top 5 significant enriched GO terms for the DEGs.

Comparisons	UP/down	GO terms	Corrected P-value
S3-0/S1-0	UP	proteasome complex	1.31E-08
		proteasome core complex	3.27E-08
		cellular iron ion homeostasis	1.59E-05
		cellular transition metal ion homeostasis	1.59E-05
		iron ion homeostasis	1.59E-05
	Down	serine-type endopeptidase activity	3.61E-06
		proteolysis	3.56E-05
		serine-type peptidase activity	5.84E-05
		serine hydrolase activity	5.84E-05
		peptidase activity, acting on L-amino acid peptides	0.00012568
S3-1/S1-1	UP	immune response	0.000653
		immune system process	0.000653
		antigen processing and presentation	0.005695
		MHC protein complex	0.0239
		MHC class II protein complex	0.0239
	Down	oxidoreductase activity	4.60E-25
		oxidation-reduction process	2.37E-20
		catalytic activity	4.52E-20
		single-organism metabolic process	4.56E-15
		serine-type endopeptidase activity	3.32E-14
S3-3/S1-3	UP	G-protein coupled receptor binding	3.53E-06
		chemokine activity	3.53E-06
		chemokine receptor binding	3.53E-06
		cytokine activity	1.73E-05
		cytokine receptor binding	9.19E-05
	Down	serine-type endopeptidase activity	2.81E-16
		endopeptidase inhibitor activity	6.99E-15
		endopeptidase regulator activity	6.99E-15
		serine-type peptidase activity	6.99E-15
		serine hydrolase activity	6.99E-15
S3-5/S1-5	UP	oxygen binding	9.28E-07
		heme binding	0.016744
		tetrapyrrole binding	0.021006
	Down	NAD+ ADP-ribosyltransferase activity	2.14E-05
		transferase activity, transferring pentosyl groups	0.001779
		chemokine activity	0.003354
		chemokine receptor binding	0.003354
		cytokine activity	0.003485

**Figure 2:**
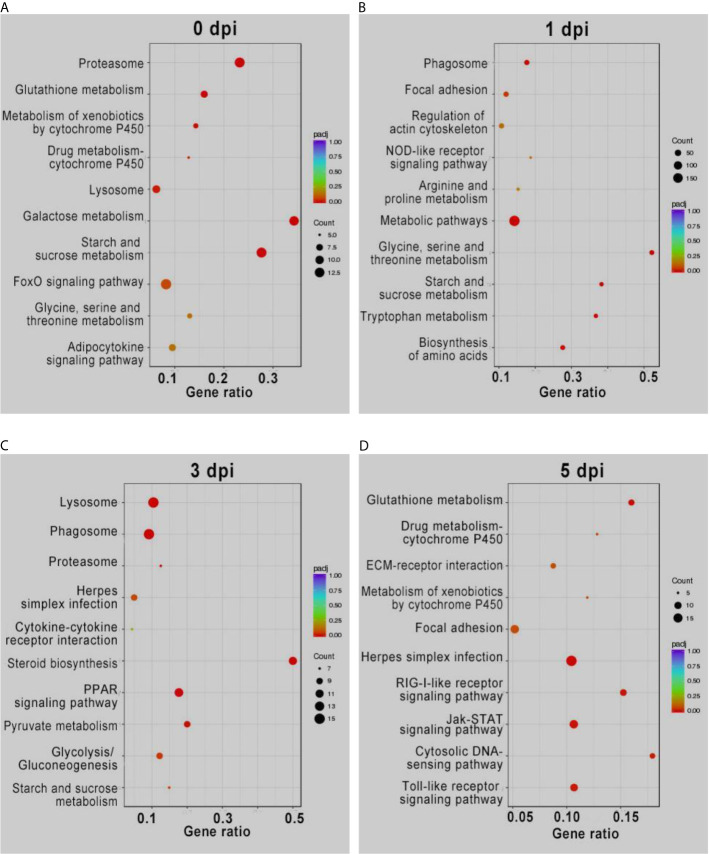
KEGG enrichment analysis of DEGs from intergroup comparisons before **(A)** and at 1 **(B)**, 3 **(C)**, and 5 **(D)** days after GCRV infection. In each time points, the upper five terms indicated the terms enriched in upregulated genes, while the bottom five terms represented the terms enriched in downregulated genes.

KEGG enrichment analysis of the DEGs showed similar results (**Figure 2**). The pathways involved in proteasome, lysosome, glutathione metabolism, and drug metabolism were significantly enriched for the upregulated DEGs before GCRV infection, while downregulated DEGs were enriched in metabolism-related pathways (galactose, starch, and sucrose metabolism) (**Figure 2A**). At 1 and 3 dpi, pathways participating in immune response and phagocytosis, such as NOD-like receptor signaling pathways, phagosomes, lysosomes, and proteasomes, were significantly enriched for the upregulated DEGs. The biosynthesis-and metabolism-related pathways were enriched for the downregulated DEGs (**Figures 2B, C**). Finally, metabolism-related pathways, such as glutathione metabolism and drug metabolism-cytochrome P450, were enriched for upregulated DEGs, while immune-related signaling pathways were enriched for the downregulated DEGs (**Figure 2D**). The results of KEGG enrichment further supported that TYO fish could respond rapidly to virus infection by initiating the immune response at the early stage of infection, whereas FMO fish had blunted immune responses that were only activated at the late stage of infection. The top five enriched KEGG terms (up and downregulated) at each time point are shown in **Figure 2**, and full details of the KEGG terms given in **Table S6**.

### Expression Patterns of DEGs in the Immune Related GO or KEGG Terms

**Figure 3:**
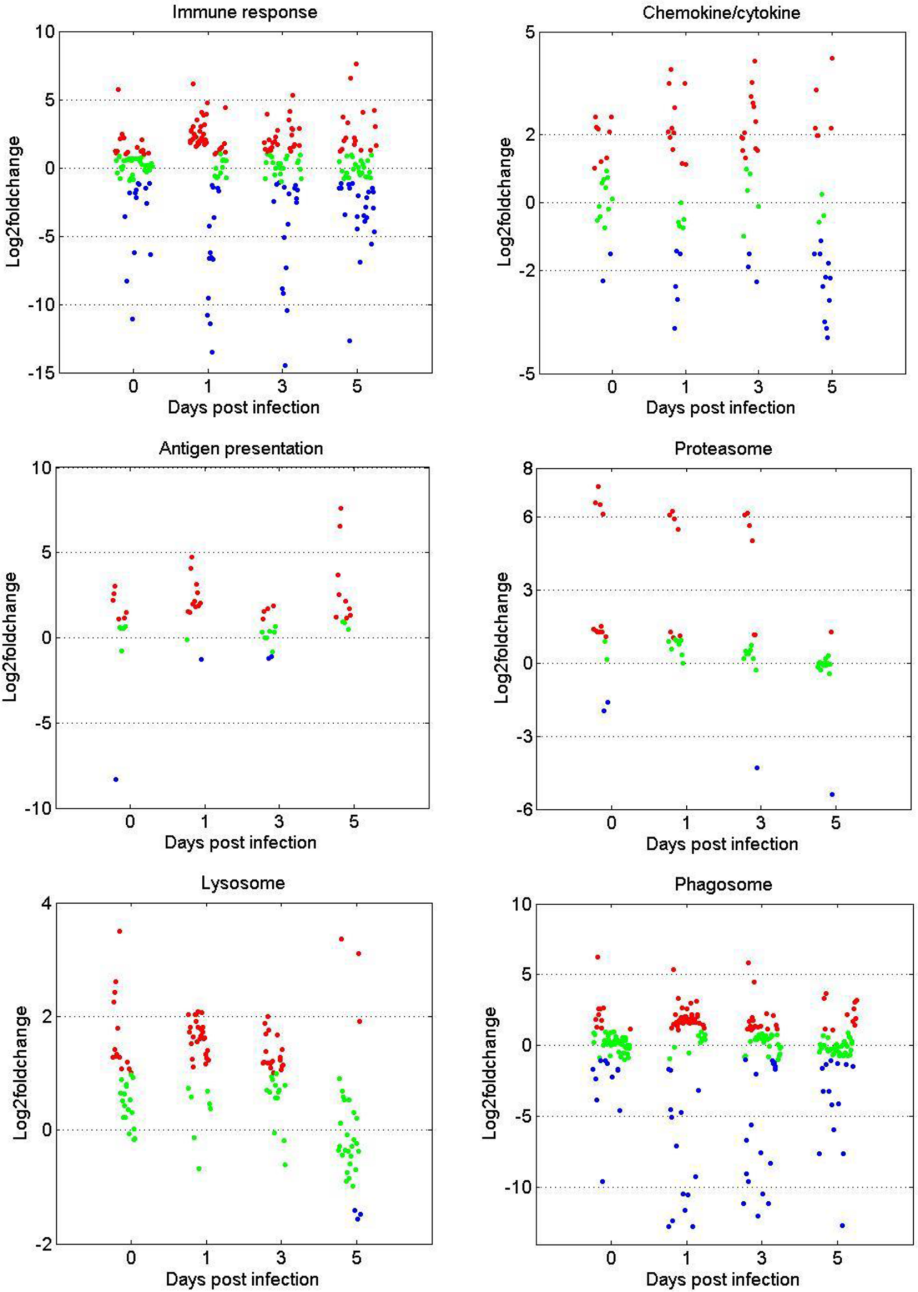
Scatterplots of gene expression pattern of DEGs in the immune related GO or KEGG terms. Scatterplots showing the log2fold change values of DEGs from intergroup comparisons in GO or KEGG terms that involved in immune response, chemokine/cytokine, antigen presentation, proteasome, phagosome, and lysosome. The red dots indicated DEGs with log2foldchange ≥ 1, the green dots represented DEGs with |log2foldchange| ≤ 1, and the blue dots stand for DEGs with log2foldchange ≤ -1.

The gene expression patterns of DEGs in the immune-related GO or KEGG terms (immune response, chemokine/cytokine, antigen processing and presentation, proteasome, phagosome, and lysosome) were selected for further analysis. As shown in **Figure 3**, in terms of immune response and chemokine/cytokine, most of the genes showed no significant difference before GCRV infection between the two age groups. However, more than half of the genes were upregulated at 1 and 3 dpi in the TYO fish group, whereas this trend ceased at 5 dpi. The genes involved in antigen presentation were almost upregulated throughout the entire infection process. For the terms proteasome, lysosome, and phagosome, over half of the genes showed upregulated expression patterns at 0, 1, and 3 dpi, but no difference was observed at 5 dpi. Moreover, we performed RT-qPCR for eight genes participating in immune-related biological processes in order to confirm the reliability of the RNA-seq data. As shown in **Supplementary Figure 1**, most genes showed expression patterns similar to those obtained by RNA-seq, although the relative expression levels were not completely consistent (**Supplementary Figure 1**). Collectively, these results show that most genes in the immune-related terms or pathways were upregulated in the TYO fish group before (0 d), or at the early stage (1 and 3 d) of GCRV infection, indicating their contribution to the age-dependent viral susceptibility in grass carp.

### Metabolome Analysis of Grass Carp Age Groups Before and After Viral Challenge

To further elucidate the mechanism of age-dependent susceptibility to GCRV in grass carp, a widely targeted metabolome analysis was performed on samples collected from the two groups before (0 d) and after GCRV infection (1, 3, and 5 d). The sample names were consistent with those in RNA-seq, and three duplicates were also performed for each sample. A total of 516 metabolites, including carbohydrates, amino acids, amino acid derivatives, fatty acids, carnitines, nucleotide derivatives (purine and pyrimidine derivatives), phospholipids (lysophosphatidylcholine [lysoPC] class, lysophosphatidylethanolamine [lysoPE] class), organic acid derivatives, oxidized lipids, benzene and substituted derivatives, and vitamins, were identified in all samples (**Table S7**). The two score plots of the principal component analysis (PCA) model show a clear separation of samples from the two age groups, indicating the difference between two groups (**Supplementary Figure 2**). Moreover, samples before GCRV infection also separated significantly from GCRV-infected samples in both groups (**Supplementary Figure 2**), implying the efficiency of GCRV infection. OPLS-DA was employed to identify differentially expressed metabolites (DEMs). Data from the TYO fish group (S3-0, S3-1, S3-3, and S3-5) were compared with data from the FMO fish group (S1-0, S1-1, S1-3, and S1-5) at the same time points. Intergroup comparisons showed that 55, 158, 190, and 190 DEMs were upregulated, whereas 67, 15, 26, and 11 DEMs were downregulated at 0, 1, 3, and 5 dpi, respectively (**Supplementary Figure 3A**). Moreover, intragroup comparisons between samples before and after GCRV infection were also done. The results showed that 146, 151, and 175 DEMs were identified in the FMO fish group (**Supplementary Figure 3B**), while 88, 107, and 110 DEMs were identified in the TYO fish group (**Supplementary Figure 3C**) at 1, 3, and 5 dpi, respectively. Detailed information on these DEMs is shown in **Table S8**. Interestingly, both inter and intragroup comparisons showed that most DEMs were upregulated in the TYO fish group and downregulated in the FMO fish group after virus infection. These results suggest that the TYO fish responded positively to virus infection, resulting in the upregulation of most metabolites, while the FMO fish were severely affected by virus infection, inducing the downregulation of most metabolites.

### Enrichment Analysis of DEMs From Intergroup and Intragroup Comparisons

**Figure 4:**
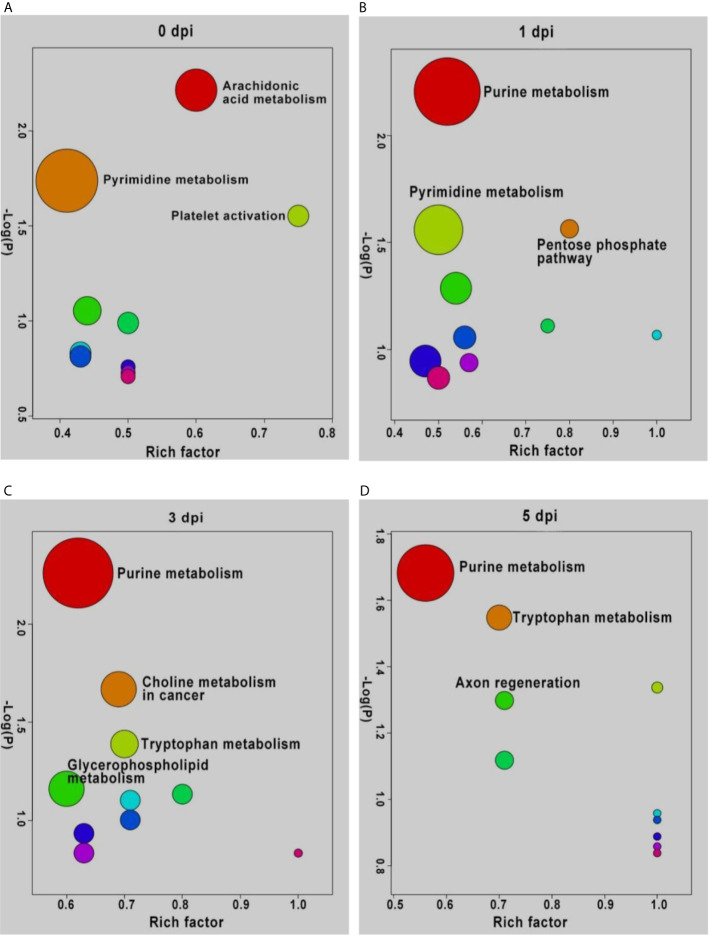
KEGG enrichment analyses of DEMs and the top ten significant DEMs from from intergroup comparisons before **(A)** and at 1 **(B)**, 3 **(C)**, and 5 **(D)** days after GCRV infection. In each time points, the top ten enriched terms were shown as dots. The size of dots indicated the enriched metabolite number and the labeled dots represented the significant enriched KEGG terms (p < 0.05).

To understand the functions of DEMs and the biological processes related to GCRV infection, all DEMs from intergroup comparisons were mapped to terms in the KEGG database. As shown in **Figure 4**, before GCRV infection, KEGG terms arachidonic acid metabolism, pyrimidine metabolism, and platelet activation were enriched (**Figure 4A**). Three metabolism-related terms (purine metabolism, pentose phosphate pathway, and pyrimidine metabolism) were enriched at 1 dpi (**Figure 4B**). At 3 dpi, four metabolism-related terms (purine metabolism, choline metabolism in cancer, tryptophan metabolism, and glycerophospholipid metabolism) were also enriched (**Figure 4C**). Finally, KEGG terms purine metabolism, tryptophan metabolism, and axon regeneration were enriched for the DEMs from 5 dpi (**Figure 4D**). Many of the enriched terms were involved in carbohydrate, nucleotide, and amino acid metabolism, indicating the different metabolism patterns between fish of different ages after virus infection. The enriched KEGG terms for the DEMs are shown in **Table S9**.

DEMs from intragroup comparisons were subjected to KEGG enrichment analysis. For the FMO fish groups, the KEGG terms choline metabolism in cancer and glycerophospholipid metabolism were significantly enriched at all time-points after GCRV infection (**Table 2**). Because most of the DEMs were downregulated in FMO fish, we propose that the two pathways were inhibited in FMO fish after GCRV infection. For the TYO fish, arachidonic acid metabolism was the most significantly enriched KEGG term after GCRV infection, followed by serotonergic synapse, inflammatory mediator regulation of TRP channels, and purine metabolism at different time points (**Table 2**). These pathways may have been activated because most DEMs in this group were upregulated after GCRV infection. These results further support different metabolic patterns between fish of different ages.

**Table 2:** KEGG enrichment analysis of DEMs from intragroup comparisons.

Groups	Dpi (dpi)	KEGG terms	P value
Five months group	1 dpi	Choline metabolism in cancer	0.001423
		Glycerophospholipid metabolism	0.006047
	3 dpi	Choline metabolism in cancer	0.00058
		Glycerophospholipid metabolism	0.002626
	5 dpi	Choline metabolism in cancer	0.0004
		Glycerophospholipid metabolism	0.002411
		Pyrimidine metabolism	0.028428
Three years group	1 dpi	Arachidonic acid metabolism	0.00017
		Serotonergic synapse	0.015474
		Inflammatory mediator regulation of TRP channels	0.015474
		Purine metabolism	0.029674
	3 dpi	Arachidonic acid metabolism	0.004301
		Purine metabolism	0.076058
	5 dpi	Arachidonic acid metabolism	0.000963
		Propanoate metabolism	0.001838
		Antifolate resistance	0.0195
		Purine metabolism	0.021864
		Pyruvate metabolism	0.030814
		Serotonergic synapse	0.038322
		Inflammatory mediator regulation of TRP channels	0.038322
		Lysosome	0.044187
		Phototransduction	0.044187
		C-type lectin receptor signaling pathway	0.044187

### Identification of Significant DEMs Between Two Groups

**Figure 5:**
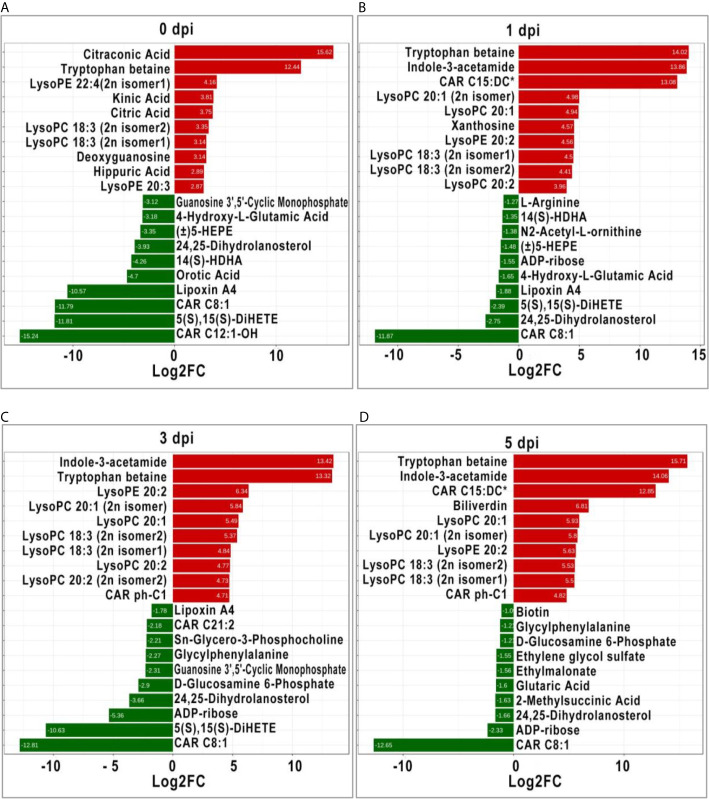
The top ten significant DEMs (up-regulated and down-regulated) from from intergroup comparisons before **(A)** and at 1 (B), 3 **(C)**, and 5 **(D)** days after GCRV infection. The red bars represented the up-regulated metabolites while the green bars indicated the down-regulated between intergroup comparisons.

It is generally recognized that more significant DEMs may play an important role in response to stimulation; therefore, the top 10 significant DEMs (upregulated and downregulated) from intergroup comparisons at each time point were identified. Before GCRV infection, the top 10 upregulated DEMs were mainly classified into three classes: organic acid and its derivatives (citraconic acid and kinic acid), amino acid metabolomics (tryptophan betaine, citric acid, and hippuric acid), and glycerophospholipids (lysoPE 22:4 [2n isomer1], lysoPC 18:3 [2n isomer2], lysoPC 18:3 [2n isomer1], and lysoPE 20:3). The top 10 downregulated DEMs were divided into two classes: carnitines (carnitine [CAR] C12:1-OH, and CAR C8:1), oxidized lipid or other lipid (5S,15S-dihydroxy-6E,8Z,10Z,13E-eicosatetraenoic acid [5(S),15(S)- DiHETE], lipoxin A4, 14S-hydroxy-4Z,7Z,10Z,12E,16Z,19Z-docosahexaenoic acid [14(S)-HDHA], 24,25-Dihydrolanosterol, and (±)-5-hydroxy-6E, 8Z,11Z,14Z,17Z -eicosapentaenoic acid [(±)5-HEPE]) (**Figure 5A**). At 1 dpi, for the top 10 upregulated DEMs, most of them were glycerophospholipids (lysoPC 20:1 [2n isomer], lysoPC 20:1, lysoPE 20:2, lysoPC 18:3 [2n isomer1], lysoPC 18:3 [2n isomer2], and lysoPC 20:2), while the others were tryptophan betaine, Indole-3-acetamide, CAR C15:DC*, and xanthosine. The top 10 downregulated DEMs were divided into three classes: oxidized lipid or other lipids (24,25-Dihydrolanosterol, 5(S),15(S)-DiHETE, lipoxin A4, (±)5-HEPE, and 14(S)-HDHA), amino acid metabolomics (4-Hydroxy-L-Glutamic acid, N2-Acetyl-L-ornithine, and L-arginine), and others (CAR C8:1 and ADP-ribose) (**Figure 5B**). Most of the top 10 upregulated DEMs at 3 dpi were also glycerophospholipids, followed by Indole-3-acetamide, tryptophan betaine, and CAR ph-C1. The top 10 downregulated DEMs were CAR C8:1, 5(S),15(S)-DiHETE, ADP-ribose, 24,25-Dihydrolanosterol, d-glucosamine 6-Phosphate, guanosine 3’,5’-cyclic monophosphate, glycylphenylalanine, Sn-Glycero-3-Phosphocholine, CAR C21:2, and LipoxinA4 (**Figure 5C**). For the last period (5 dpi), half of the top 10 upregulated DEMs were also glycerophospholipids (lysoPC and lysoPE), while the others were carnitines (CAR C15:DC* and CAR ph-C1), tryptophan betaine, Indole-3-acetamide, and biliverdin. The top 10 downregulated DEMs contained organic acid derivatives (2-Methylsuccinic acid, glutaric acid, ethylmalonate, and ethylene glycol sulfate) and others (CAR C8:1, ADP-ribose, 24, 25-Dihydrolanosterol, d-glucosamine 6-Phosphate, glycylphenylalanine, and biotin) (**Figure 5D**). Apparently, glycerophospholipids (lysoPC and lysoPE) were significantly upregulated in TYO fish at all time-points, indicating it is an important role in defense against virus infection.

### DEMs Related to the Metabolism of Carbohydrates, Amino Acids, Glycerophospholipids, and Nucleotides

**Figure 6:**
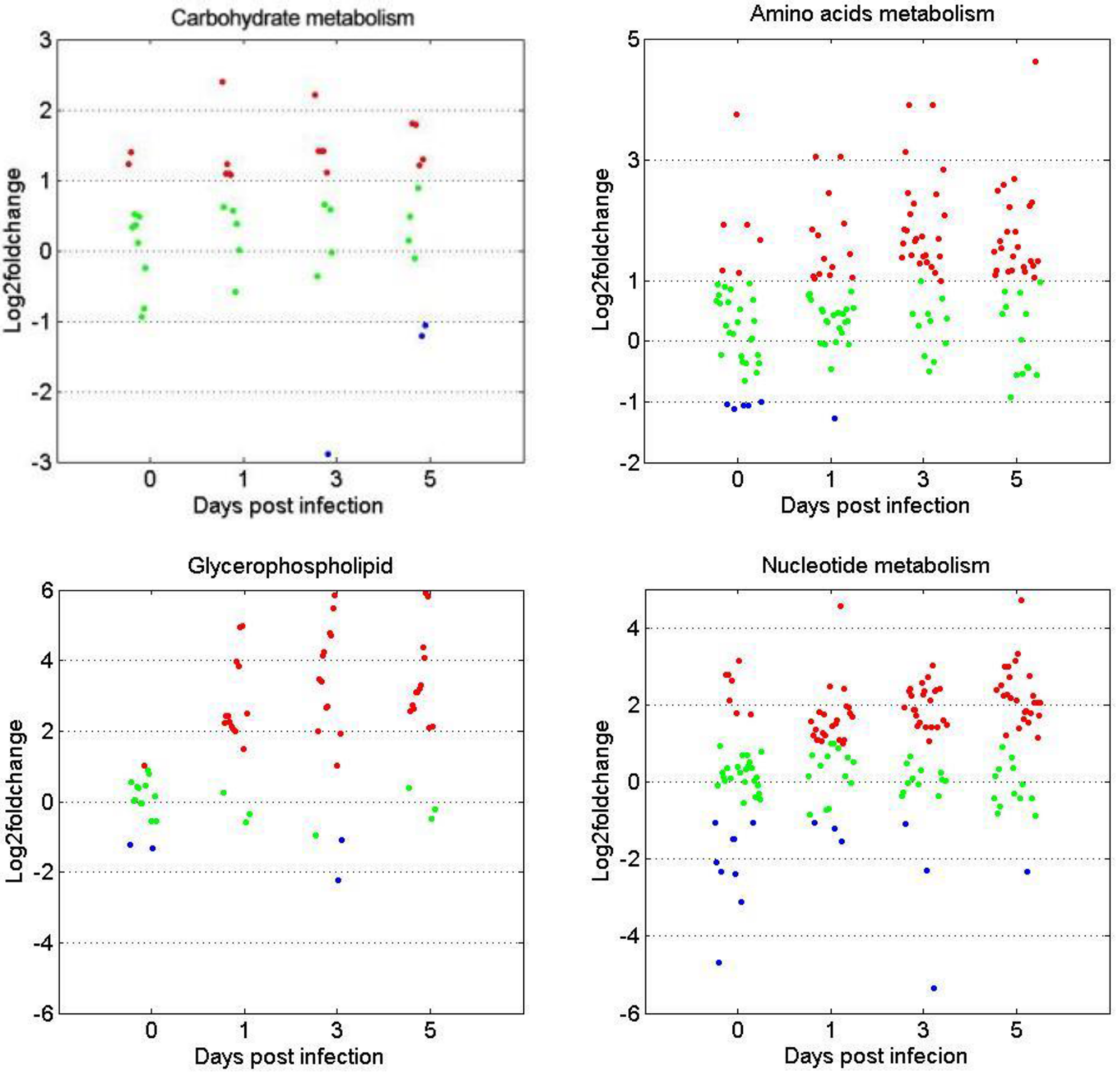
Scatterplots of gene expression pattern of DEMs in the reprehensive metabolism pathways. Scatterplots showing the log2fold change values of DEMs from intergroup comparisons in in the reprehensive metabolism pathways (carbohydrate metabolism, amino acids metabolism, glycerophospholipids, and nucleotides metabolism). The red dots indicated DEMs with log2fold change ≥ 1, the green dots represented DEGs with |log2foldchange| ≤ 1, and the blue dots stand for DEGs with log2foldchange < -1.

The expression patterns of metabolites involved in the metabolism of carbohydrates, amino acids, glycerophospholipids, and nucleotides were compared between the two age groups. **Figure 6** shows that for the four metabolite categories, most of them showed no significant difference between the two groups before GCRV infection (**Figure 6**). However, more than half of them were upregulated at 1, 3, and 5 dpi in the TYO fish, with only a few being downregulated. The upregulation of metabolites in TYO fish indicated their ongoing transcription, translation, and biosynthesis to defend against viral infection, whereas in FMO fish, the downregulation of metabolites indicated that the host translation machinery was hijacked by the virus, resulting in death.

### The Anti-Viral Effects of DEMs

**Figure 7:**
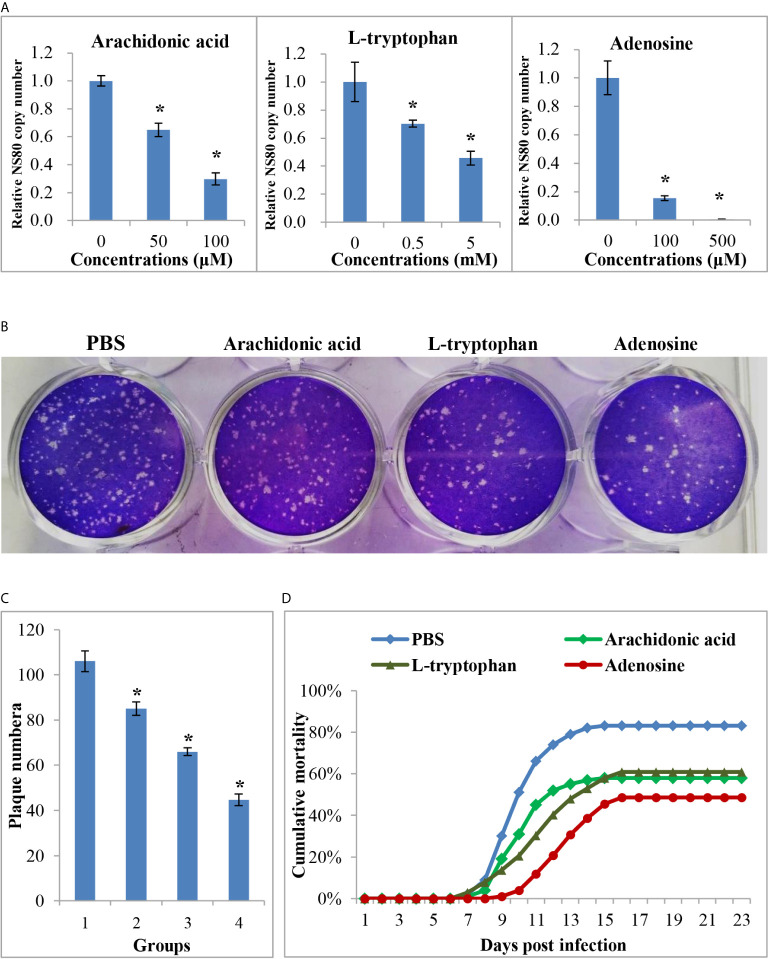
The anti-viral effects of differential expressed metabolites. **(A)** The relative copy number of GCRV nonstructural protein gene NS80 in different metabolites treated cells or in untreated cells. **(B)** Plaque assay of different metabolites treated cells or in untreated cells. **(C)** The plaque numbers of different groups that calculated from three biological duplications. 1: PBS treated group; 2: arachidonic acid treated group; 3: L-tryptophan treated group; 4: adenosine treated group. **(D)** Survival curve of different metabolites treated or PBS treated fish after GCRV infection. Significant difference (P < 0.05) between the control and treated groups was indicated with asterisks (*).

The above results showed that many metabolites were differentially expressed between the two age groups, whereas their role during virus infection was unclear. Three DEMs, arachidonic acid, L-tryptophan, and adenosine, were selected for further analysis. The CCK-8 assay indicated that the three metabolites showed no cytotoxicity at concentrations of 100 μM, 5 mM, and 500 μM, respectively (**Supplementary Figure 4**). Therefore, cells were treated with three metabolites at these concentrations to investigate their role during viral infection. As shown in **Figure 7**, all three metabolites inhibited GCRV replication in a dose-dependent manner, especially adenosine. RT-qPCR revealed that the copy number of NS80 and VP7 in metabolite-treated cells was significantly lower than that in untreated cells (**Figure 7A** and **Supplementary Figure 4**). The plaque assay also showed that the number of plaques in the metabolite-treated cells was significantly less than that in the untreated cells (**Figures 7B, C**). Moreover, to further investigate the role of metabolites during virus infection *in vivo*, FMO grass carp were injected with different doses of metabolites or the same volume of PBS (control group) and then subjected to viral challenge experiments. **Figure 7D** shows that all three metabolites reduced the mortality of grass carp after GCRV infection, whereas PBS did not. Specifically, the mortality rates in the arachidonic acid, L-tryptophan, and adenosine injected groups were 58.0%, 60.1%, and 48.5%, respectively, while mortality in control group was up to 83.0%. Collectively, these results indicate the antiviral effects of differentially expressed metabolites.

## Discussion

The grass carp is an important farmed fish in China, accounting for approximately 16% of global freshwater aquaculture. Their significant economic value is threatened by their susceptibility to viral infections, has raised concerns among scientists (14, 24–26). Before this study, it was known that grass carp showed age-dependent susceptibility to GCRV, with those less than one year old considered susceptible, and those older than three years of age resistant. However, the mechanisms causing this discrepancy remained poorly understood. In this study, we compared susceptible FMO fish with resistant TYO fish using different methods to elucidate the mechanisms underlying this phenomenon.

### Immune Response

It is well known that the immune response plays an important role in host defense against pathogen invasion. Properly regulated immune responses can eliminate invading pathogens, while a disordered or immoderate immune response can lead to organism damage (14, 27, 28). The transcriptome sequencing results from this study are particularly interesting. At 0, 1, and 3 dpi, the immune-related terms, including proteasome, lysosome, phagosome, antigen processing and presentation, and chemokine/cytokine activity were enriched in upregulated genes of TYO fish. Moreover, other terms, such as glutathione metabolism, iron ion homeostasis, and drug metabolism-cytochrome P450, were also enriched in upregulated genes at the same time points. It is known that glutathione plays important roles in antioxidant defense, cell proliferation and apoptosis, signal transduction, cytokine production, and immune responses and iron ion homeostasis is important for host defense against pathogen infection (29–32). The expansion of the cytochrome P450 gene family was accounted for the koala’s ability to detoxify eucalyptus foliage (33). The upregulation of these terms, combined with the immune-related terms in the TYO fish group, suggests that the fish could recognize the virus rapidly and then initiate the immune response to eliminate the invading virus. Nevertheless, the immune-related pathways were not activated until 5 dpi in the younger fish group, implying that the immune response was repressed to benefit virus replication. Therefore, these results may be one of the reasons for age-dependent susceptibility to GCRV.

### Carbohydrate and Amino Acid Metabolism

Carbohydrates are the main energy source for cells and other life processes. Amino acids play several roles, including functioning as building blocks of proteins and taking part in the synthesis of ATP and metabolites with various biological functions (34, 35). Many of the metabolites related to carbohydrate and amino acid metabolism showed no significant difference in expression levels between the two fish groups before GCRV infection. Additionally, most DEMs, especially the metabolites involved in amino acid metabolism, were upregulated in the TYO fish group. Of relevance is a study on virus-infected olive flounder fish, whereby it was found that amino acid metabolism was suppressed in viral hemorrhagic septicemia for the biosynthesis of viral proteins (36). Another study found that glucose was the most crucial biomarker between survival and death in tilapia, and that glucose enhances their defense against *Edwardsiella tarda* infection through metabolome reprogramming (37, 38). Considering that the TYO fish were resistant while FMO fish were susceptible to GCRV, we proposed that TYO fish respond to virus infection effectively, resulting in the upregulation of carbohydrates and amino acids to provide materials and energy for improved defense against the virus. Nevertheless, the FMO fish failed to defend against virus infection and the cell components may be utilized by viruses for viral protein synthesis, leading to the downregulation of metabolites involved in carbohydrate and amino acid metabolism.

### Glycerophospholipid Metabolism

Metabolomics showed that the glycerophospholipid metabolism pathway was activated in TYO fish and repressed in FMO fish. The expression patterns of DEMs related to glycerophospholipid metabolism were upregulated in TYO fish. Glycerophospholipids are ubiquitous in nature and play a crucial role as components of cellular membranes or subcellular organelle membranes (39). Moreover, glycerophospholipids act as binding sites for intracellular and intercellular proteins and are involved in metabolism and signaling (40). Coincidently, we also observed cell necrosis in the spleen of FMO fish, indicating that the cell membranes were broken in FMO fish after virus infection, resulting in the downregulation of the glycerophospholipid metabolism pathway. Nevertheless, the activation of pathways related to membrane-structure organelles (proteasome, lysosome, and phagosome) in TYO fish indicated the formation of membrane-structured organelles to eliminate the virus. Therefore, these results highlight the important role of glycerophospholipids in host defense against viral infections.

### Nucleotide Metabolism

The nucleotide metabolism-related pathways (pyrimidine metabolism and purine metabolism) were activated in TYO fish after virus infection, and DEMs related to these pathways were mainly upregulated in this group. Nucleotides are central to biological signaling and the transfer of genetic information, which are essential for DNA and RNA synthesis, and therefore, for protein synthesis (41, 42). The upregulation of these pathways in TYO fish may be due to them responding positively to virus infection and the initiation of DNA replication, RNA transcription and translation, as well as protein synthesis, in order to eliminate the virus. The downregulation of these pathways in FMO fish implies that the host translation machinery is hjjacked or shut down by GCRV to facilitate the replication and spread of the virus. Similarly, the nucleotide metabolism-related pathways were downregulated in classical swine fever virus-infected piglets (43), and purine metabolism was downregulated in bisphenol A-treated zebrafish (44, 45). Collectively, these results show the vital role of nucleotide metabolism in response to virus infection or toxicity stimulation.

### Arachidonic Acid Metabolism

We found that the arachidonic acid metabolism pathway was also significantly upregulated in TYO fish after virus infection. Arachidonic acid is a polyunsaturated omega-6 fatty acid and a precursor in the biosynthesis of prostaglandins, thromboxanes, and leukotrienes. Additionally, arachidonic acid has been reported to act as a key inflammatory intermediate and play an important role in the immune response (46). As found for carbohydrate and amino acid metabolism, virus-infected olive flounder fish had increased levels of arachidonic acid with viral hemorrhagic septicemia, suggesting an inflammatory response occurred (36). Arachidonic acid also showed an elevated trend in the serum of adult human patients with primary dengue infection (47), and in hepatitis C-infected tree shrews, metabolomic analysis revealed that arachidonic acid is one of the most significant differential metabolites (48). Therefore, the upregulation of the arachidonic acid metabolism pathway in TYO fish after virus infection may be responsible for the elevated immune response and inflammatory response in this group at the early stage of infection, which is beneficial for host defense against virus invasion.

**Figure 8:**
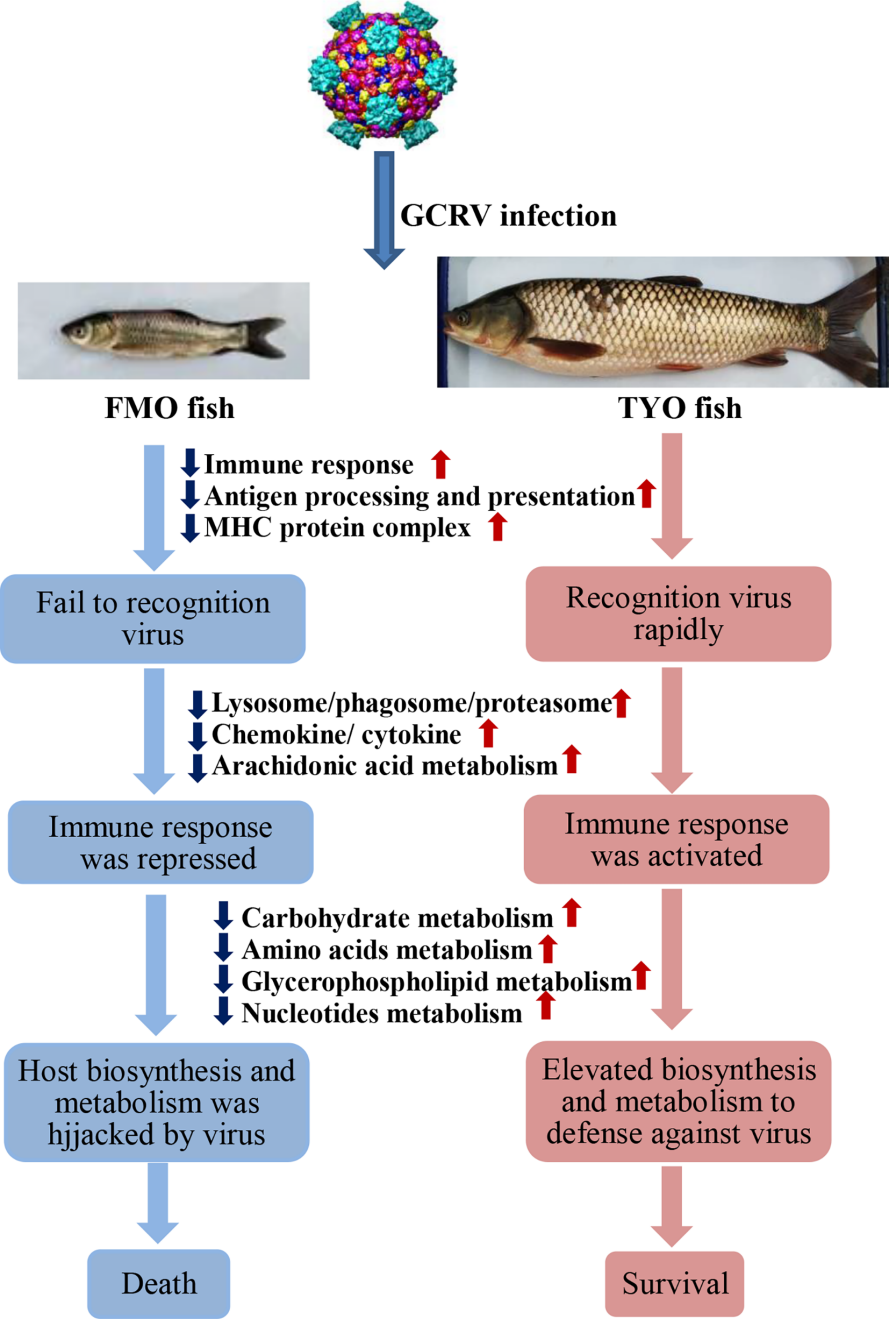
The schematic diagram of the reasons for the age-dependent viral susceptibility in grass carp. The downward dark blue arrows indicated these representative pathways were down-regulated in FMO fish groups, while the upward red arrows represented these pathways were up-regulated in TYO fish.

In summary, based on the results obtained in this study, we concluded that not only the immune system, but also host biosynthesis and metabolism, account for the age-dependent susceptibility to GCRV in grass carp. As shown in **Figure 8**, The FMO fish failed to recognize the invaded virus, failed to initiate the immune response immediately, and the host translation machinery was hijacked by the virus for viral protein synthesis, resulting in death. However, the older, TYO fish recognized the virus immediately, rapidly activated the immune response, and elevated host translation machinery involved in DNA replication, RNA transcription and translation, as well as biosynthesis and metabolism to defend against viruses (**Figure 8**).

## Data Availability Statement

The datasets presented in this study can be found in online repositories. The names of the repository/repositories and accession number(s) can be found in the article/**Supplementary Material**.

## Ethics Statement

The animal study was reviewed and approved by the committee of the Institute of Hydrobiology, Chinese Academy of Sciences.

## Author Contributions

LH, YW, and ZZ designed research. LH, DZ, XL, and YL performed research. RH, CY, and LL contributed new reagents or analytic tools. LH, DZ, and XL analyzed data. LH and YW wrote the paper. All authors contributed to the article and approved the submitted version.

## Funding

This work was supported by the National Natural Science Foundation of China (NO. 32073017 and 31702322 to LH), the Strategic Pilot Science and Technology Projects (A) Category of CAS (No. XDA24030203 to YW), the Youth Innovation Promotion Association CAS (No. 2021338 to LH), and State Key Laboratory of Freshwater Ecology and Biotechnology (NO. 2019FBZ05 to YW).

## Conflict of Interest

The authors declare that the research was conducted in the absence of any commercial or financial relationships that could be construed as a potential conflict of interest.
