# Supplementary figures and images for: Multi-Omics Sequencing Provides Insights Into Age-Dependent Susceptibility of Grass Carp (Ctenopharyngodon idellus) to Reovirus
Source: Front Immunol. 2021 Jun 17;12:694965. doi: 10.3389/fimmu.2021.694965 (PMC8247658; doi:10.3389/fimmu.2021.694965)

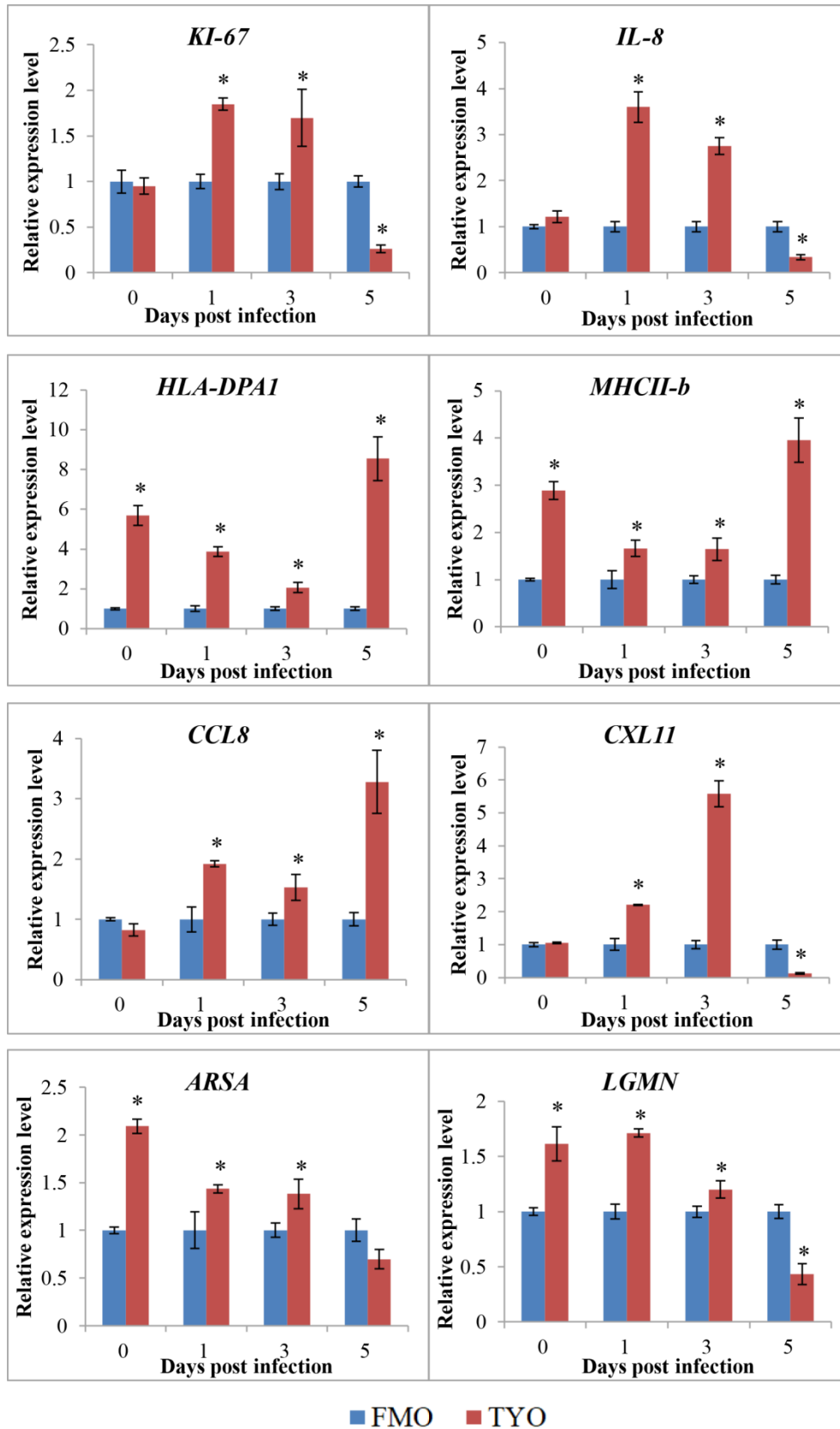

**Supplementary Figure 1** Expression pattern of 8 genes involved in immune Response.

Supplement: Supplementary file 1 [file Image_1.pdf]
