# Supplementary material for: Multi-Omics Sequencing Provides Insights Into Age-Dependent Susceptibility of Grass Carp (Ctenopharyngodon idellus) to Reovirus
Source: Front Immunol. 2021 Jun 17;12:694965. doi: 10.3389/fimmu.2021.694965 (PMC8247658; doi:10.3389/fimmu.2021.694965)
Supplement: Supplementary file 2 [file Image_2.pdf]

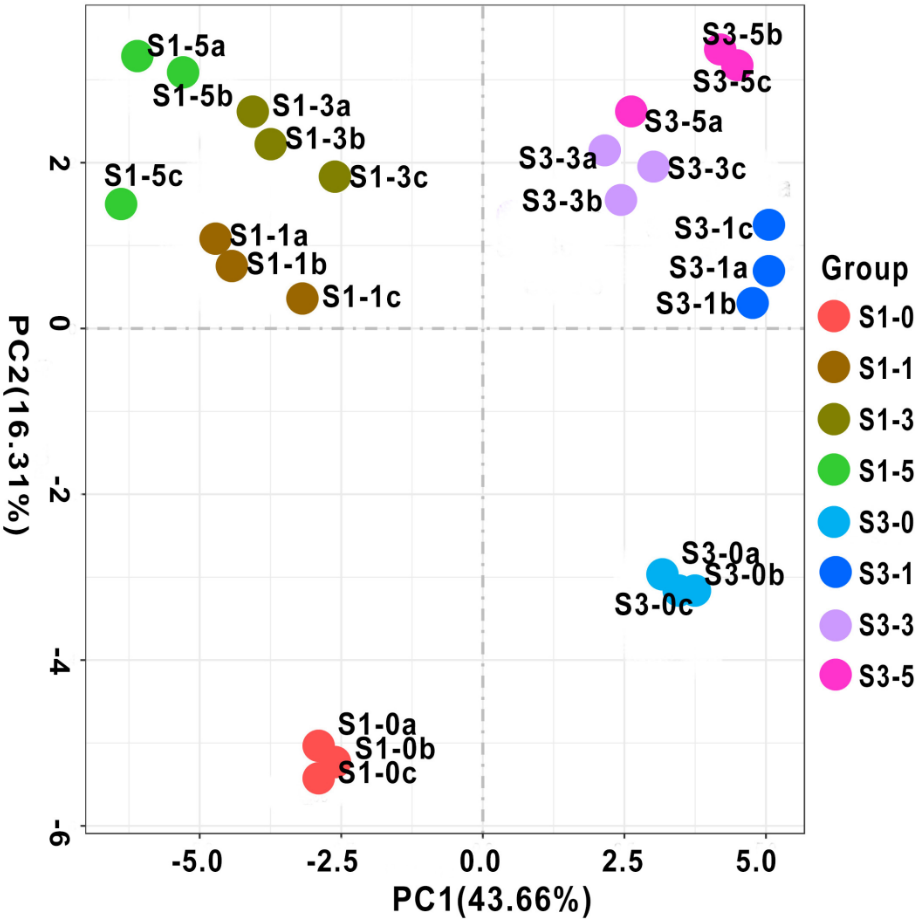

Supplementary Figure 2 PCA score plots for the metabolomics profile of samples from different aged groups. In the plot, PC1 showed the difference between the samples from different groups, whereas PC2 revealed the difference between infected and uninfected samples.
