# Supplementary material for: Multi-Omics Sequencing Provides Insights Into Age-Dependent Susceptibility of Grass Carp (Ctenopharyngodon idellus) to Reovirus
Source: Front Immunol. 2021 Jun 17;12:694965. doi: 10.3389/fimmu.2021.694965 (PMC8247658; doi:10.3389/fimmu.2021.694965)
Supplement: Supplementary file 3 [file Image_3.pdf]

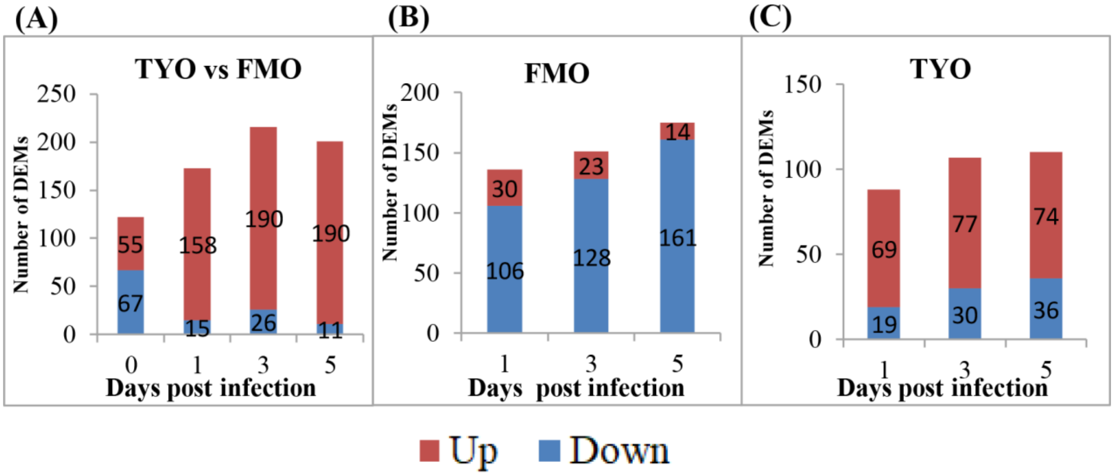

Supplementary Figure 3 The differentially expressed metabolites (DEMs) in different comparisons. (A) The DEMs that identified in intergroup comparison at before and after GCRV infection. (B) The DEMs that identified in TYO fish at different time points after GCRV infection. (C) The DEMs that identified in FMO fish at different time points after GCRV infection. The red patterns represented the up-regulated DEMs whereas the blue patterns indicated the down-regulated DEMs.
