# Supplementary material for: Multi-Omics Sequencing Provides Insights Into Age-Dependent Susceptibility of Grass Carp (Ctenopharyngodon idellus) to Reovirus
Source: Front Immunol. 2021 Jun 17;12:694965. doi: 10.3389/fimmu.2021.694965 (PMC8247658; doi:10.3389/fimmu.2021.694965)
Supplement: Supplementary file 4 [file Image_4.pdf]

(A)

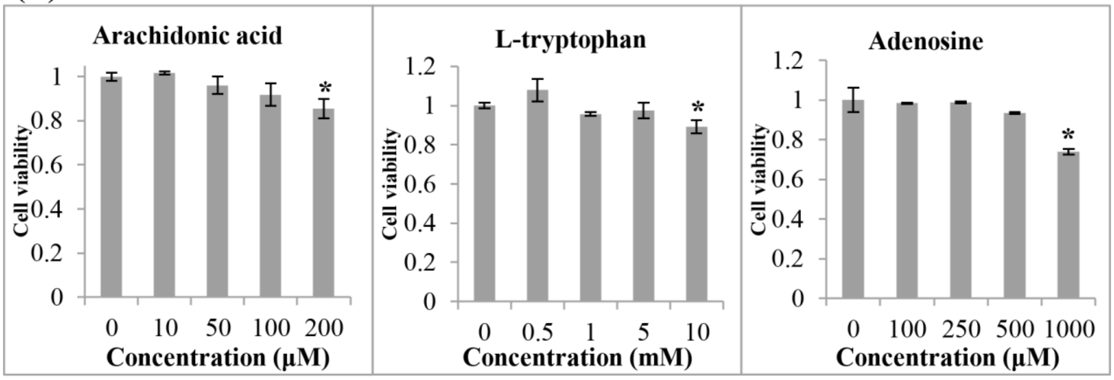

(B)

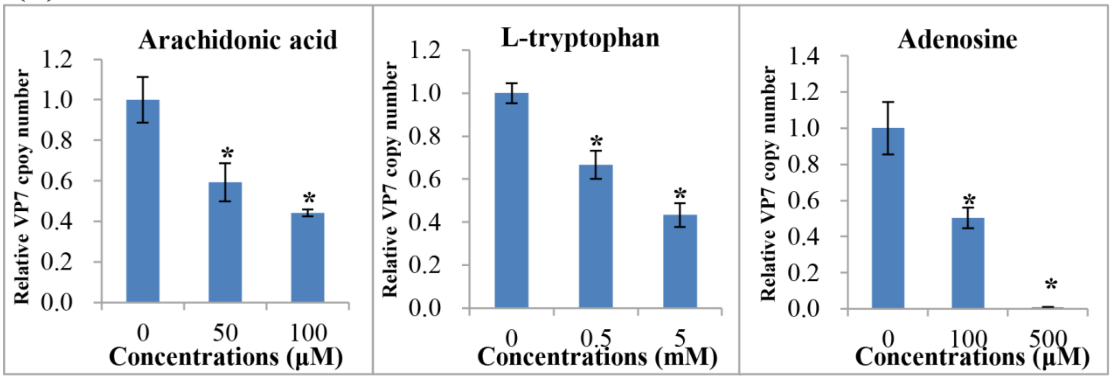

Supplementary Figure 4 The anti-viral effects of differential expressed metabolites. (A) CCK-8 analysis the cytotoxicity of different metabolites. (B) The relative copy number of GCRV structural protein gene VP7 in different metabolites treated cells or in untreated cells.
