# Supplementary material for: Multi-Omics Sequencing Provides Insights Into Age-Dependent Susceptibility of Grass Carp (Ctenopharyngodon idellus) to Reovirus
Source: Front Immunol. 2021 Jun 17;12:694965. doi: 10.3389/fimmu.2021.694965 (PMC8247658; doi:10.3389/fimmu.2021.694965)
Supplement: Supplementary file 5 [file Table_1.docx]

**Table S1 Primer sequences for qPCR analysis**

| **Target genes** | **Primer sequences (5’-3’)** | **efficiency** |
| --- | --- | --- |
| *KI-67* | CTCGCAGTGATGTAGAGACTGATACTG  GCCTTGATTATCTCCCCCTCG | 91.2% |
| *IL-8* | ATTACTGAAGCAATGAGTCTTAGAGGTC  TTGGTTTCCTTCAGGGTGGC | 94.0% |
| *HLA-DPA1* | TTATGAACTCAGTGTTAGTGAAATGGC  GATGGAGGGTAGAAGCCAGTCAC | 98.9% |
| *MHCII-b* | CATCGTTGTTACAGCAATATAGGGC  TGATTGGTTGAGGGTAGAAGTCGT | 90.7% |
| *CCL8* | CCTTTTTTCTGTAACAATTTGGACTGT  TGATTCCACCCCCTTCCATTC | 90.3% |
| *CXL11* | CTTGCCTGCCTGATCGCTG  GCATTTCCGTCCTGCACCAT | 94.0% |
| *ARSA* | TGTTTTACTACCCCATTGACCCA  GGGTCTGCCTCCAGGTTAAATAG | 94.4% |
| *LGMN* | GCTGAAAAGCGGTCCAAATGA  TAATGTCAACAGGCAGAGGCTTC | 94.1% |
| *NS80* | GTCACTGCCCATTGCTTCGC  GCGGCACGGGATCTGTTGTA | 96.3% |
| *VP7* | CGCACCTGTGACTTGGACGA  AGCGAGTCAGCACCTTGTCG | 98.7% |
| *β-actin* | AGCCATCCTTCTTGGGTATG  GGTGGGGCGATGATCTTGAT | 99.2% |
