# Supplementary material for: Multi-Omics Sequencing Provides Insights Into Age-Dependent Susceptibility of Grass Carp (Ctenopharyngodon idellus) to Reovirus
Source: Front Immunol. 2021 Jun 17;12:694965. doi: 10.3389/fimmu.2021.694965 (PMC8247658; doi:10.3389/fimmu.2021.694965)
Supplement: Supplementary file 6 [file Table_2.docx]

| **Sample name** | **duplicates** | **Raw reads** | **Clean reads** | **Clean bases** | **Error rate(%)** | **Q20(%)** | **Q30(%)** |
| --- | --- | --- | --- | --- | --- | --- | --- |
| S1_0 | a | 44086572 | 43593782 | 6.54 | 0.03 | 95.35 | 87.87 |
|  | b | 43959191 | 42775222 | 6.42 | 0.03 | 96.48 | 89.53 |
|  | c | 46024032 | 44762682 | 6.71 | 0.03 | 96.26 | 89.13 |
| S1_1 | a | 51010200 | 50514496 | 7.58 | 0.03 | 97.28 | 92.48 |
|  | b | 43638964 | 42449807 | 6.36 | 0.03 | 95.79 | 88.08 |
|  | c | 45255896 | 44724541 | 6.7 | 0.03 | 95.6 | 87.67 |
| S1_3 | a | 54323458 | 53607662 | 8.04 | 0.03 | 97.41 | 92.81 |
|  | b | 44397927 | 43195411 | 6.47 | 0.03 | 96.21 | 89.14 |
|  | c | 43318566 | 42425322 | 6.36 | 0.03 | 96.32 | 89.42 |
| S1_5 | a | 55571282 | 54586266 | 8.19 | 0.03 | 97.29 | 92.59 |
|  | b | 45354222 | 44337729 | 6.65 | 0.03 | 96.02 | 88.58 |
|  | c | 44613776 | 43405844 | 6.5 | 0.03 | 96.13 | 88.83 |
| S3_0 | a | 56858808 | 56037244 | 8.41 | 0.03 | 97.18 | 92.3 |
|  | b | 43771968 | 42577304 | 6.38 | 0.03 | 96.24 | 89.2 |
|  | c | 45897450 | 44780684 | 6.72 | 0.03 | 96.19 | 89.02 |
| S3_1 | a | 46107132 | 45499748 | 6.82 | 0.03 | 97.55 | 93.18 |
|  | b | 45463769 | 44240714 | 6.64 | 0.03 | 96.23 | 89.2 |
|  | c | 43539923 | 42385696 | 6.36 | 0.03 | 96.27 | 89.11 |
| S3_3 | a | 47414770 | 46790432 | 7.02 | 0.03 | 97.5 | 93 |
|  | b | 43359534 | 42206217 | 6.33 | 0.03 | 96.33 | 89.25 |
|  | c | 44268235 | 43179075 | 6.48 | 0.03 | 96.38 | 89.33 |
| S3_5 | a | 53585926 | 52903280 | 7.94 | 0.03 | 97.43 | 92.82 |
|  | b | 44763427 | 43596861 | 6.54 | 0.03 | 96.3 | 89.19 |
|  | c | 42440949 | 41278911 | 6.19 | 0.03 | 96.23 | 89.1 |

**Table S2 Summary of sequencing data in the study**
