# Supplementary material for: Multi-Omics Sequencing Provides Insights Into Age-Dependent Susceptibility of Grass Carp (Ctenopharyngodon idellus) to Reovirus
Source: Front Immunol. 2021 Jun 17;12:694965. doi: 10.3389/fimmu.2021.694965 (PMC8247658; doi:10.3389/fimmu.2021.694965)
Supplement: Supplementary file 7 [file Table_3.docx]

**Table S3 Summary of DEGs in intergroup comparisons**

| **Days post**  **infection** | **Comparisons** | **Up** | **Down** | **Total** |
| --- | --- | --- | --- | --- |
| 0 | S3-0/S1-0 | 300 | 569 | 869 |
| 1 | S3-1/S1-1 | 898 | 1040 | 1938 |
| 3 | S3-3/S1-3 | 393 | 555 | 948 |
| 5 | S3-5/S1-5 | 428 | 724 | 1152 |
